# Strengths, Weaknesses, Opportunities, and Challenges of Conditional Cash Transfers Under the Janani Shishu Suraksha Karyakram in India: A Narrative Review

**DOI:** 10.7759/cureus.82874

**Published:** 2025-04-23

**Authors:** Urvish Joshi, Tejas Shah, Vaidehi Gohil, Rachit Y Sharma, Venu R Shah

**Affiliations:** 1 Community Medicine, Narendra Modi Medical College and Sheth Lallubhai Gordhandas Municipal General Hospital, Ahmedabad, IND; 2 Community Medicine, Gujarat Medical Education and Research Society Medical College, Patan, IND; 3 Community Medicine, Dr Mansukh Kanjibhai Shah Medical College and Research Center, Ahmedabad, IND; 4 Internal Medicine, Gujarat Medical Education and Research Society Medical College and Hospital, Ahmedabad, IND; 5 Community Medicine, Gujarat Cancer Society Medical College, Hospital and Research Center, Ahmedabad, IND

**Keywords:** a review, india, janani shishu suraksha karyakram, maternal and child health, swot analysis

## Abstract

Background: Janani Suraksha Yojana (JSY), a scheme launched by the central government of India, aimed to reduce maternal mortality by incentivizing institutional deliveries through conditional cash transfers (CCTs). An expansion to this initiative, the Janani Shishu Suraksha Karyakram (JSSK), incorporates free maternal and neonatal services. However, despite reported progress in improving access, questions remain about program efficiency and equity. This review synthesizes evidence on the strengths, weaknesses, opportunities, and challenges (SWOC) of the JSY/JSSK CCT component of the scheme.

Methods: A narrative review of 19 studies on the cash transfer component of the Janani Shishu Suraksha Karyakram (JSSK), published between 2009 and 2025, was conducted using PubMed, Embase, and Google Scholar. Studies were thematically analyzed to assess CCT-related implementation, equity, quality of care, and health outcomes.

Results: Strengths include increased institutional deliveries and improved access among marginalized populations. Weaknesses involve payment delays, persistent out-of-pocket expenses, and uneven quality of care. Opportunities include expanding incentives across the continuum of care, leveraging technology, and integrating with other schemes like PMMVY and Namo Shree Yojana. Challenges include regional disparities, implementation variability, and uncertainty around long-term behavioral change.

Conclusion: While the JSY/JSSK CCT component has improved service uptake, its full potential requires stronger implementation, inter-scheme coordination, and quality assurance. Policymakers should prioritize equity, accountability, and integration to enhance maternal and newborn health outcomes.

## Introduction and background

Launched in 2005, the Janani Suraksha Yojana (JSY) is a significant national health initiative launched by the central government of India aimed at decreasing maternal mortality by eliminating out-of-pocket expenses for institutional deliveries [1-3]. This initiative emphasizes facility-based care to enhance safety during the critical period around childbirth. In 2011, Janani Shishu Suraksha Karyakram (JSSK) was launched as an expansion of JSY by offering a more comprehensive continuum of care. In JSSK, benefits were extended to include sick infants up to one year of age and to provide free treatment for prenatal and postnatal complications. A crucial element of JSSK's strategy is the use of conditional cash transfers (CCTs), primarily administered through the Janani Suraksha Yojana (JSY) [2]. The CCT approach reduces financial obstacles and encourages pregnant women, particularly those from disadvantaged backgrounds, to opt for institutional deliveries, ultimately aiming to improve key maternal and child health outcomes, specifically by reducing maternal and infant mortality and morbidity.

Research indicates that the program has played a crucial role in reducing health disparities by increasing the rates of institutional deliveries among at-risk groups [4,5]. Nevertheless, despite these achievements since its launch, the program still faces ongoing challenges. Key issues include inefficiencies in implementation, such as payment delays, documentation obstacles, and low awareness, as well as resource leakage [6]. Furthermore, beneficiaries often continue to face out-of-pocket costs, which contradicts the program's goal of offering free services [7,8].

Although the expanded JSSK initiative offers a broad range of complimentary services, this narrative review focuses specifically on the evidence related to its CCT component. The aim is to compile existing research coming out of India, on the strengths, weaknesses, opportunities, and challenges (SWOC) of this CCT aspect, utilizing significant studies published from 2009 to 2025. This review specifically explores how well the conditional cash transfer component of the program has contributed to achieving its core goals-namely, reductions in maternal and neonatal mortality, and improvements in access to quality maternal healthcare. The results are intended to provide valuable insights for policymakers, public health professionals, and researchers who are looking to improve the effectiveness of CCTs in national health initiatives. 

## Review

Methodology

This narrative review employed a systematic approach to gather evidence on the SWOC of CCTs under JSY and its successor, JSSK. A SWOC (Strengths, Weaknesses, Opportunities, and Challenges) framework was adopted for this review due to its capacity to synthesize multifaceted evidence from both quantitative and qualitative studies. Unlike trend analyses or purely outcome-based categorizations, the SWOC method enables a comprehensive evaluation of program implementation, contextual barriers, and potential policy improvements, thus offering a more integrated understanding of the CCT component within the broader maternal and child health landscape. A total of 19 studies were included in the review by following the PRISMA framework to ensure transparency, reproducibility, and methodological rigor.

A thorough literature search was conducted using three major electronic databases: PubMed, Embase, and Google Scholar. The search strategy aimed to identify significant research articles published in English from January 2009 to March 2025, specifically focusing on the Indian context. Key search terms included "Janani Shishu Suraksha Karyakram", "JSSK", "conditional cash transfers", "cash transfers", and "India", which were combined using Boolean operators (AND, OR) to ensure a comprehensive and relevant search.

The inclusion criteria for articles were: (1) original research articles, systematic reviews, and meta-analyses focusing on the CCT aspect of JSSK (primarily JSY); (2) studies conducted within the Indian context; (3) publication in peer-reviewed journals indexed in the specified databases; and (4) publication within the designated time-frame (2009-2025). Studies that concentrated solely on the broader JSSK program without specific analysis of the CCT component were excluded. The scope for inclusion of study designs was kept broad for both qualitative and quantitative study designs. Conference proceedings and opinion pieces were generally excluded, editorials or commentaries with qualitative analysis that followed a scientific methodology were included, and relevant information from government reports and reputable organizations was considered to provide context.

**Figure 1:**
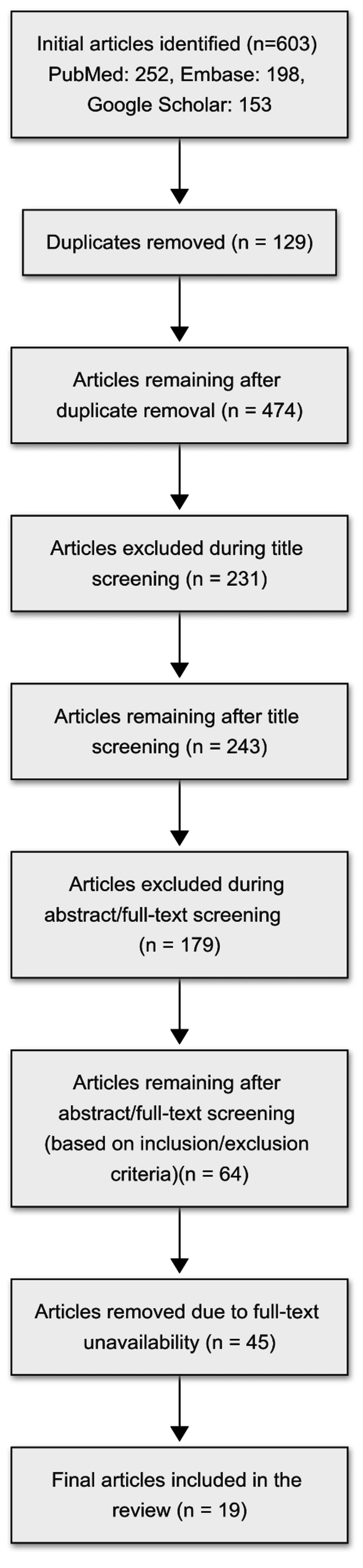
Flow of evidence screening

The initial search yielded numerous records, which were screened by examining titles and abstracts for relevance, and duplicates were removed using bibliographic management software [9]. Searched articles were evaluated against inclusion and exclusion criteria. Next, efforts were made to obtain as many full texts as possible from the remainder. A manual search of reference lists was conducted to find other relevant studies in order not to miss out on worthy evidence (Figure 1).

Three reviewers (TS, VG, RS) independently screened and evaluated all identified articles. Any discrepancies in their assessments were resolved through discussion or, if consensus could not be reached, by adjudication from a fourth reviewer (UJ). Data extraction was done by two authors (UJ and VS). A predefined template was used to gather information on study design, methodology, and key findings related to SWOCs of the CCT component, as well as regional differences and effects on maternal and child health outcomes. Data synthesis involved thematic analysis to identify recurring themes and patterns concerning the SWOC of the program. To enhance transparency and clarity, study-wise findings have been summarized along with critical appraisal, which aligns individual study contributions with specific SWOC categories and provides quality appraisal using standardized tools.

Results

The SWOC analysis of CCTs under India's JSSK program is the main focus of this narrative review of 19 studies within the Indian context, published between 2010 and 2025, with a particular focus on the JSY, the program's predecessor. The reviewed studies primarily employed a range of methodologies, including ecological studies (n=2), quantitative analyses (n=2), qualitative studies (n=2), statistical impact evaluation (n=1), review/analysis (n=1), replication study (n=1), observational and qualitative study (n=1), vignette-based survey (n=1), cross-sectional facility and community-based study (n=1), community-based descriptive cross-sectional study (n=1), replication and extension study (n=1), general cross-sectional study (n=1), re-estimation of causal impacts (n=1), spatial analysis (n=1), quasi-experimental cross-sectional study (n=1), and cross-sectional community-based study (n=1).

**Table 1:** Characteristics of studies included in the review SWOC: Strengths, Weakness, Opportunities, Challenges; JSY: Janani Suraksha Yojana, NRHM: National Rural Health Mission; JSSK: Janani Shishu Suraksha Karyakram; OOPE: out-of-pocket expenditure; PHC: primary health center; RMC: Respectful Maternity Care; PCMC scale: Person-Centered Maternity Care scale

Authors	Year of Publication	Study Design	Study Setting/Region	Sample Size	Intervention/Program Focus	Key Outcomes Measured	Key Findings Relevant to SWOC
Lim et al. [10]	2010	Statistical impact evaluation	India (national level)	Not specified for all analyses	JSY, a conditional cash transfer programme	Institutional delivery, skilled birth attendance, antenatal care, maternal, perinatal, and neonatal mortality	The study found significant positive effects of JSY on antenatal care, institutional delivery, and skilled birth attendance. In two of three analytic approaches, they also found a reduction in perinatal and neonatal deaths, but not in the district-level difference-in-differences approach.
Nair and Panda [11]	2011	Review/analysis	India	Not applicable	Quality of maternal healthcare in India and the impact of the NRHM (which includes JSY)	Various indicators of maternal healthcare quality (not specified in detail in excerpt)	Discusses the quality of maternal healthcare and suggests that while NRHM has increased access, issues with quality remain a significant challenge.
Randive et al. [12]	2013	Ecological	Nine states in India	284 districts (aggregated)	JSY, a conditional cash transfer programme	Institutional delivery and Maternal Mortality Ratio (MMR)	Documented steep rises in institutional delivery proportions since JSY began, but the extent to which JSY has succeeded in raising institutional deliveries across different socioeconomic subgroups needs further investigation, pointing to a potential weakness in equitable reach.
Randive et al. [13]	2014	Ecological	Nine states in India	284 districts	JSY, a conditional cash transfer programme	Institutional birth proportions and Maternal Mortality Ratio (MMR)	The study aimed to investigate the association between institutional birth proportions (after JSY initiation) and MMR, contributing to understanding the program's potential impact (strength or opportunity). It also highlighted the need for further investigation in low-resource settings (challenge).
Carvalho et al. [14]	2014	Replication study	India (national level, re-examining Lim et al., 2010)	Not explicitly stated (uses data from DLHS surveys)	JSY, a conditional cash transfer programme	Institutional delivery, skilled birth attendance, antenatal care, and maternal, perinatal, and neonatal mortality	The study aimed to replicate the findings of Lim et al. (2010) and assess their robustness. Identifying coding issues and variations in results suggests potential weaknesses in the original study and opportunities for more rigorous analysis.
Chaturvedi et al. [15]	2014	Observational and qualitative study	Three districts in Madhya Pradesh, India	18 deliveries observed, followed by interviews with staff	Quality of obstetric care in the context of the JSY program.	Observed practices of nurse-midwives during deliveries and their perspectives on providing care.	Findings indicated inadequate knowledge and skills among nurse-midwives, leading to limited monitoring and potentially compromising the quality of care despite increased institutional births (a weakness or challenge).
Chaturvedi et al. [16]	2015	Vignette-based survey	Three heterogeneous districts in Madhya Pradesh, India	233 nurses	Highlighted inadequate clinical competence among nurse-midwives managing deliveries under JSY, suggesting that increased institutional deliveries driven by CCTs may not consistently ensure quality care—a key weakness in implementation.	Competence scores in managing hemorrhage and eclampsia	Found poor levels of competence among nurse-midwives in managing obstetric complications, which could explain the slow decline in maternal mortality despite increased institutional births (a weakness or challenge).
Thongkong et al. [17]	2017	Quantitative analysis	Five districts in Jharkhand and Odisha states, India	14,184 women (with a birth in the last year)	JSY coverage and equity	Institutional deliveries and receipt of JSY benefits	Found evidence of pro-rich inequalities in institutional deliveries and JSY benefit receipt, indicating a weakness in the program's ability to reach the poorest and an opportunity to improve equity.
Salve et al. [18]	2017	Cross-sectional facility and community-based study	Primary Health Centre, Chhainsa, Ballabgarh, New Delhi, India	All deliveries in the facility over 32 months (specific number not in excerpt)	Impact of JSSK implementation	Number of deliveries conducted in the facility	The study compared the number of deliveries before and after JSSK implementation in the study area, likely aiming to assess the program's effectiveness in increasing institutional births (potential strength).
Chaudhary et al. [7]	2017	Community-based, descriptive, cross-sectional study	PHC, Lakhan Majra, Haryana, India	200 women who delivered between July 2013 and September 2014	Utilization of JSY	Awareness and utilization of JSY benefits	The study assessed the awareness and utilization of JSY among women in the study area, which could reveal strengths or weaknesses in program outreach and uptake at the community level.
Sharma et al. [19]	2018	Qualitative study	A district in Haryana, India	50 postnatal women	Ground-evel implementation of conditional cash transfer scheme (likely JSY, though not solely focused)	Experiences and perceptions of beneficiaries regarding the scheme	Highlighted challenges faced by beneficiaries in accessing scheme benefits, such as difficulties in arranging documents and low benefit amounts in some areas, thus indicating weaknesses in implementation and program design.
Gupta et al. [20]	2018	Qualitative study	Three Empowered Action Group (EAG) states in India	68 health workers and program officials	Implementation of JSY at the grassroots and intermediary levels	Perspectives of stakeholders on the preparedness and constraints faced by the JSY	Identified several barriers to effective JSY implementation, including issues with human resources, infrastructure, drug supply, and referral systems, representing significant weaknesses and challenges.
Carvalho and Rokicki [21]	2019	Replication and extension study	India (national level, re-examining Lim et al., 2010)	189,533 (after corrections in exact matching analysis)	JSY, a conditional cash transfer programme	Probabilities of in-facility delivery, skilled birth attendance, and neonatal mortality rates	Found the original authors’ results to be replicable and robust. Multilevel models revealed meaningful heterogeneity across states and districts in JSY's effects, highlighting opportunities for geographically targeted improvements and challenges of uneven implementation.
Yangala et al. [22]	2020	Cross-sectional study	Tertiary care hospital, Telangana, India	228 postnatal women	JSSK utilization and OOPEs	Utilization of JSSK components and OOPEs during childbirth and neonatal illnesses	Found high utilization of JSSK services but significant out-of-pocket expenditure on transport, indicating a weakness in the scheme's ability to fully eliminate costs and an opportunity to address transport coverage.
Aizawa [23]	2021	Re-estimation of causal Impacts	India (national level)	Not specified	JSY, a conditional cash transfer programme	Institutional delivery, skilled birth attendance, antenatal care, tetanus toxoid injections, and intakes of iron and folic acid supplements	Identified potential selection bias and insufficient control for unobserved confounders in earlier JSY evaluations, indicating a methodological weakness in existing evidence. The study also presents an opportunity to strengthen future evaluations through improved econometric strategies and more robust causal inference.
Mishra et al. [24]	2021	Spatial analysis	India (national level, using NFHS-4 data)	148,145 women (last birth in an institution)	JSY service utilization	JSY service utilization (institutional delivery with JSY benefit)	Showed spatial inequality in JSY service coverage across Empowered Action Group (EAG) states, indicating a weakness in universalization and a need for revisiting policy strategies at regional/district levels (challenge).
Godha et al. [4]	2022	Quantitative analysis	India (national level, using NFHS-III and NFHS-IV data)	34,036 (NFHS-III), 189,143 (NFHS-IV)	JSY and related state-specific government schemes (conditional cash transfers for institutional delivery)	Three or more antenatal (ANC) visits; institutional delivery (ID); and postnatal care (PNC) within 2 days of delivery	The study investigated changes in inequality in maternal healthcare service uptake over a decade, offering insights into the strengths or weaknesses of JSY in promoting equitable access.
Chatterjee and Poddar [25]	2023	Quasi-experimental, cross-sectional, difference-in-difference study	India (national level)	India Human Development Survey (IHDS) - II data from 2011-12	JSY, conditional cash transfers for institutional delivery	Children's standardized test scores (reading, writing, math)	JSY shows strengths in positive educational spillovers and women's empowerment, weaknesses in potential for increased fertility, opportunities to address multiple developmental issues, and challenges in fiscal constraints.
Kaur et al. [26]	2024	Cross-sectional, community-based study	Ballabgarh block, Faridabad district in Haryana, India	424 pregnant women	Assessment of RMC during childbirth	Women's experiences of respectful and disrespectful care during childbirth using the PCMC scale	The study assessed the prevalence of disrespect and abuse during childbirth, highlighting a potential weakness in the quality of care provided in health facilities, even with programs like JSY promoting institutional delivery.

The geographical scope of these studies varies significantly, ranging from national-level analyses to regional investigations across multiple states, such as the Empowered Action Group (EAG) states, and more localized studies focused on specific districts (e.g., Madhya Pradesh, Haryana, Telangana), particular areas, municipalities, or individual medical facilities. This diversity enables a nuanced understanding of how implementation and outcomes differ across settings. There is also a great deal of variation in sample sizes, ranging from large-scale quantitative analyses with tens or hundreds of thousands of women or deliveries to smaller qualitative studies with dozens of participants.

The main focus of the interventions and programs being examined is JSY, a CCT program created to encourage institutional deliveries; however, some studies are also assessing the larger JSSK initiative. Numerous indicators of maternal and child health, including institutional delivery rates, skilled birth attendance, antenatal care utilization, maternal mortality ratio, neonatal mortality, and associated factors like out-of-pocket expenses, care quality, and women's experiences, are among the key outcomes measured across the studies. When taken as a whole, these studies offer a thorough analysis of the JSY/JSSK programs, using a variety of methodologies to evaluate their implementation, impact, and related opportunities and challenges in the Indian healthcare setting.

The analysis of the selected literature identified several significant strengths, weaknesses, opportunities, and challenges associated with the conditional cash transfer component of the JSSK in India.

Strengths

Numerous studies have highlighted a key strength of the JSY program: a notable rise in institutional deliveries after its implementation [10,18]. This consistent outcome across different research methods and regions highlights the program's success in meeting its main goal of promoting childbirth in healthcare facilities. Lim et al. (2010) conducted the first comprehensive statistical evaluation of the program across India, demonstrating significant positive impacts on institutional deliveries [10]. These initial results have been further validated by replication studies, such as those by Carvalho and Rokicki (2018), which used robustness checks and additional model specifications to reassess the impact of financial incentives, confirming the increase in institutional births [14,21]. Women frequently mentioned that the cash incentive was crucial in making institutional delivery more accessible by covering transportation expenses and compensating for lost wages during childbirth. This underscores the program's effectiveness in improving financial accessibility and reducing barriers to essential maternal health services.

Additionally, the CCT component has enhanced healthcare utilization beyond delivery, leading to higher rates of childhood immunization, antenatal care visits, and postnatal check-ups [14]. Carvalho et al. (2014) discovered that financial assistance from JSY resulted in increased immunization rates, ranging from a 3.1% rise for one dose of the polio vaccine to a 9.1% increase in the proportion of fully vaccinated children [14]. Their research also showed that JSY led to higher post-partum check-up rates and healthier early breastfeeding practices around childbirth [14]. This indicates a positive spillover effect of the program, influencing a wider range of maternal and child health behaviors.

One of the program's key strengths is its ability to lessen health disparities, particularly by significantly boosting institutional deliveries among women from economically disadvantaged, less educated, and rural backgrounds [4]. Godha and Hotchkiss (2022) examined shifts in wealth-related inequality concerning the use of institutional delivery and other maternal health services, discovering a notable reduction in inequality for institutional delivery both nationally and in states with varying performance levels [4]. Qualitative studies have emphasized that the program has been especially advantageous for marginalized groups who previously encountered substantial obstacles in accessing institutional care. A study by Randive et al. highlighted a decrease in access inequality since the JSY's inception [13]. This underscores the program's effectiveness in reaching underserved populations and narrowing healthcare access gaps.

Emerging evidence also points to possible long-term benefits across generations, such as enhanced educational outcomes for children in beneficiary households [25]. Chatterjee and Poddar (2023) reported significant positive spillovers of JSY on children's educational achievements, noting improvements in reading, math, and writing scores due to increased human capital investments in these families [25]. This suggests that the positive effects of the CCT may extend beyond immediate health outcomes, potentially influencing the future well-being of families.

Weaknesses

Although the program has achieved some success, it also has notable shortcomings. Key issues include challenges in implementation and leakages, with evidence indicating that the most impoverished individuals do not always receive the intended benefits. This is due to problems such as payment delays, difficulties in acquiring eligibility documents, and a lack of awareness. A study by Sharma et al. (2018) highlighted issues like the untimely release of funds, challenges in obtaining Below Poverty Line (BPL) cards, insufficient awareness of the scheme, and banking illiteracy among recipients [19]. Many beneficiaries expressed frustration with bureaucratic hurdles and delays in receiving cash transfers, pointing to significant bureaucratic obstacles and delays that hinder access.

The emphasis on institutional delivery might overshadow the broader importance of continuous maternal care, with less notable improvements in the uptake and equity of ANC and PNC [23]. Godha and Hotchkiss (2022) found that while institutional delivery achieved significant reductions in inequality, the progress in ANC and PNC uptake and inequality was less pronounced, highlighting the need to focus more on comprehensive maternal care [4]. This indicates that the limited scope of the incentive may not be effectively promoting all aspects of maternal healthcare.

Methodological issues include the possibility of selection bias and uncontrolled confounding factors in some evaluation studies. A study re-evaluating the causal impacts of JSY suggests that previous analyses may not have fully accounted for selection bias through observable characteristics [23]. This raises concerns about the reliability of some reported impact estimates.

One significant flaw is the continued out-of-pocket expenses for beneficiaries, even though the program is designed to offer free services [7]. In a study by Chaudhary et al. (2017) conducted in a rural region of Haryana, it was discovered that 83.5% of participants faced out-of-pocket costs, with a median expense of 1100 rupees, often due to the absence of facilities like ultrasound at public health centers. Women reported spending on transportation, food, and occasionally even medications within healthcare facilities, highlighting substantial hidden costs and financial burdens despite the program's objectives.

The varying effects on mortality outcomes reported in different studies raise questions about the program's success in meeting its primary objective of reducing maternal and neonatal deaths [10,12]. Although Lim et al. (2010) identified a decrease in perinatal and neonatal deaths in some analyses, they found no effect on maternal mortality [10]. Additionally, Carvalho and Rokicki's (2018) replication study highlighted significant differences across states and districts regarding the impact of JSY on mortality outcomes [14,21]. This inconsistency indicates that merely increasing institutional deliveries may not be enough to substantially lower mortality rates without also addressing factors such as the quality of care.

Opportunities

There are several methods to enhance the program's efficiency. Expanding incentives to include ANC and PNC can strengthen the continuum of care, which may result in more significant improvements in maternal and child health [4]. Focusing solely on institutional delivery could miss crucial opportunities for earlier intervention during pregnancy and the postnatal period.

Enhancing equity can be achieved by improving the targeting and minimizing leakages through more accurate identification of beneficiaries and more efficient processes. It is essential to ensure that cash transfers reach the intended recipients promptly and without unnecessary bureaucratic obstacles to maximize the program's impact on the most vulnerable groups.

Utilizing technology for registering beneficiaries, executing direct cash transfers, and spreading health information holds considerable promise. Mobile platforms and digital payment systems have the potential to boost efficiency, minimize delays, and improve transparency within the program.

Enhancing community participation and strengthening the role of ASHAs can significantly improve program acceptance and effectiveness. Accredited Social Health Activists (ASHAs) play a vital role in connecting with pregnant women within communities and can be further empowered to provide enhanced support and advice, thereby boosting both awareness and the use of services. In terms of out-of-pocket expenditures, healthcare providers should ensure adequate information is available, particularly regarding transportation-related concerns, by integrating JSSK's CCT component with other health and social programs, the multifaceted issues impacting maternal and child health can be tackled [22]. Collaborating with initiatives that emphasize nutrition, sanitation, and women's empowerment could create synergistic effects, leading to more comprehensive improvements in health outcomes.

Challenges

The program encounters several ongoing obstacles. A primary issue is financial sustainability, given the significant expenditure needed to run a program of this magnitude on a national level [4]. Securing continuous funding and effectively allocating resources are crucial for the long-term success of JSSK.

Equity issues and regional differences in both the implementation and outcomes continue to exist due to the diverse contexts at the state level. In a federal structure, the disparities are influenced by the differing eligibility requirements and cash transfer amounts between states with varying performance levels, as well as differences in healthcare infrastructure and service delivery [21]. A re-evaluation of the plan is necessary to guarantee the program's universal accessibility [24]. Researchers have also pointed out in their study that the JSY scheme by itself is not enough to eliminate the gap in institutional delivery rates between wealthy and impoverished populations [17].

Even with a rise in institutional delivery rates, obstacles pertaining to access to public health services and the quality of care provided can hinder the program's success [20]. Challenges such as insufficient resources, poor infrastructure, and negative experiences reported by women at certain facilities can discourage use or compromise the quality of care. Reports of negative experiences among pregnant women, including a lack of privacy and disrespectful behavior from staff, underscore the urgent need to enhance the quality of care and patient experience [11]. Women who delivered in private health facilities were more likely to receive respectful maternity care (RMC) compared to those who delivered in government facilities [26]. Additionally, improving the skills of birth attendants in handling obstetric complications is also essential in order to increase rates of institutional deliveries under JSY and thereby reduce the maternal mortality [15,16]. The long-term sustainability of behavioral changes prompted by cash transfers remains uncertain. Although the program has effectively increased institutional deliveries, it is crucial to determine whether these changes in health-seeking behavior will continue once financial incentives are no longer provided.

Discussion

This narrative review compiles evidence regarding the strengths, weaknesses, opportunities, and challenges associated with the conditional cash transfer aspect of India's JSSK program. The studies included generally exhibit strong methodological rigor and quality. They consistently adhere to high research standards across quantitative, qualitative, and mixed-methods approaches. The Newcastle-Ottawa Scale [27,28] and Risk Of Bias In Non-randomized Studies - of Interventions (ROBINS-I) tool [29,30] effectively highlighted methodological strengths, such as adequate representativeness, reliable outcome assessments, and appropriate statistical tests, though studies commonly faced limitations due to moderate risks associated with selection biases and confounding factors inherent in observational designs. The adapted Critical Appraisal Skills Programme (CASP) checklist for qualitative studies underscored the high quality of qualitative inquiry into programmatic barriers and implementation dynamics but noted transparency gaps regarding researcher-participant relationships [31]. The Joanna Briggs Institute (JBI) checklist further affirmed the methodological soundness of systematic reviews and mixed-methods analyses, yet consistently revealed minor limitations related to confounding control and exposure measurement validity [32,33]. Collectively, these methodological assessments emphasize the overall credibility of findings while prompting careful interpretation regarding causality. Importantly, identified gaps such as inconsistent geographical coverage and limited consideration of contextual heterogeneity indicate opportunities for methodological refinement in future evaluations. This quality appraisal aligns with broader literature patterns, confirming the importance of rigorous methodological frameworks for interpreting the effectiveness and equity implications of conditional cash transfers. Future research would benefit from enhanced methodological transparency, particularly around confounding control strategies, and expanded geographic and contextual coverage to strengthen evidence-based policy recommendations for JSSK program implementation.

Overall, the evidence base is of high methodological quality, with rigorous statistical and methodological standards consistently applied across studies. Limitations primarily related to smaller sample sizes in some studies do not significantly detract from the robustness of the findings (Table 2).

**Table 2:** Critical appraisal of research articles using Newcastle-Ottawa scale (NOS) ★: Adequately meets the criterion, 0: Does not adequately meet the criterion, ★★: Exceeds expectation or particularly strong in meeting the criterion.

Study Identification	Representativeness	Sample Size	Non-Response Rate	Ascertainment Tool	Confounder Control	Outcome Assessment	Statistical Test	Overall Score
Thongkong et al. [17]	★ (Truly representative)	★ (≥400)	★ (≥95%)	★★ (Validated tool clearly described)	★ (Confounders controlled)	★★ (Reliable outcome assessment)	★ (Appropriate test)	8/10 (High)
Salve et al. [18]	★ (Truly representative)	★ (≥400)	★ (≥95%)	★★ (Validated tool clearly described)	★ (Confounders controlled)	★★ (Reliable outcome assessment)	★ (Appropriate test)	8/10 (High)
Chaudhary et al. [7]	★ (Truly representative)	0 (<400)	★ (≥95%)	★★ (Validated tool clearly described)	★ (Confounders controlled)	★★ (Reliable outcome assessment)	★ (Appropriate test)	7/10 (Moderate)
Yangala et al. [22]	★ (Truly representative)	0 (<400)	★ (≥95%)	★★ (Validated tool clearly described)	★ (Confounders controlled)	★★ (Reliable outcome assessment)	★ (Appropriate test)	7/10 (Moderate)
Godha et al. [4]	★ (Truly representative)	★ (≥400)	★ (≥95%)	★★ (Validated tool clearly described)	★ (Confounders controlled)	★★ (Reliable outcome assessment)	★ (Appropriate test)	8/10 (High)
Kaur et al. [26]	★ (Truly representative)	★ (≥400)	★ (≥95%)	★★ (Validated tool clearly described)	★ (Confounders controlled)	★★ (Reliable outcome assessment)	★ (Appropriate test)	8/10 (High)

The overall quality of the evidence base from the appraised studies is high. Each study clearly articulated its aims, employed rigorous qualitative methods, thoroughly addressed ethical considerations, and provided valuable insights for improving maternal health programs under India's JSY scheme. Minor limitations exist concerning the transparency of researcher-participant relationships. (Table 3).

**Table 3:** Critical Appraisal of Research Articles Using an Adapted CASP Qualitative Checklist CASP: Critical Appraisal Skills Programme

Study	Clear Statement of Aims	Appropriate Qualitative Methodology	Appropriate Research Design	Appropriate Recruitment Strategy	Data Collection Addressed Research Issue	Relationship Between Researcher and Participants Considered	Ethical Issues Addressed	Rigorous Data Analysis	Clear Statement of Findings	Research Value	Overall Quality
Chaturvedi et al. [15]	Yes, clear objective of assessing birth attendants' competence.	Yes, qualitative methodology (case vignettes) appropriate for assessing competence.	Yes, justified use of clinical vignettes method.	Yes, clear description of selection and rationale.	Yes, vignette-based survey method aligned closely with objectives.	Can't Tell, insufficient detail provided on researcher-participant dynamics.	Yes, ethical approval indicated clearly.	Yes, detailed scoring system explained and validated.	Yes, explicit findings clearly reported.	High, directly informs policy and practice improvements.	High
Sharma et al. [19]	Yes, clearly aims to understand implementation barriers and utilization factors.	Yes, qualitative interviews appropriately explore subjective experiences.	Yes, justified use of semi-structured interviews.	Yes, clearly described purposive sampling strategy.	Yes, clearly described semi-structured interview process.	Can't Tell, relationship and potential biases not explicitly considered.	Yes, ethical approval and confidentiality clearly mentioned.	Yes, clear inductive and deductive thematic analysis described.	Yes, detailed presentation of findings with direct participant quotes.	High, informs actionable implementation improvements.	High
Gupta et al [20]	Yes, aims to identify barriers from the supply-side perspectives.	Yes, qualitative interviews are suitable for exploring perceptions and barriers.	Yes, use of in-depth interviews across multiple levels justified.	Yes, clear description of purposive sampling across various stakeholders.	Yes, detailed explanation of interview methods and setting.	Can't Tell, researcher influence and bias not explicitly detailed.	Yes, ethical considerations clearly addressed.	Yes, thematic analysis robustly described, verified through triangulation.	Yes, clear statement of findings, supported by substantial evidence.	High, provides deep insights into structural and systemic barriers.	High

The appraised studies, primarily evaluating JSY, exhibit an overall serious risk of bias due to the inherent challenges of quasi-experimental designs in addressing confounding and selection bias. The replication study by Carvalho and Rokicki reinforces the original findings but shares similar methodological limitations [21]. The Aizawa (2021) study provides a rigorous and transparent analysis of the JSY conditional cash transfer program using advanced statistical methods [23]. While the risk of bias is low in most domains, the overall rating is moderate due to the unavoidable potential for unmeasured confounding in observational research. The evidence is credible and valuable for policy and practice, but findings should be interpreted with appropriate caution regarding causality (Table 4).

**Table 4:** Critical appraisal/risk of bias assessment of included study using the ROBINS-I tool ROBINS-I: Risk Of Bias In Non-randomized Studies - of Interventions

Study Identification	Bias due to Confounding	Bias in Selection of Participants	Bias in Classification of Intervention	Bias due to Deviations from Intended Interventions	Bias due to Missing Outcome Data	Bias in Measurement of the Outcome	Bias in Selection of the Reported Result	Overall Risk of Bias
Lim et al. [10]	Moderate	Serious risk	Low risk	Not Applicable	Unclear risk	Moderate risk	Unclear risk	Serious risk
Carvalho et al. [14]	Moderate to Serious	Serious risk	Low risk	Not Applicable	Unclear risk	Moderate risk	Low to Moderate risk	Serious risk
Carvalho and Rokicki [21]	Moderate	Serious risk	Low risk	Not Applicable	Unclear risk	Moderate risk	Unclear risk	Serious risk
Aizawa [23]	Moderate	Low	Low	Low	Low	Low	Moderate	Moderate

The overall quality of the evidence base appraised from the included studies is robust, demonstrating strong methodological rigor in quantitative analyses and sound qualitative exploration. Limitations were minor and primarily related to controlling for confounders and ensuring the comprehensive validity of exposure measures (Tables 5-6).

**Table 5:** Critical appraisal of studies using the relevant Joanna Briggs Institute (JBI) checklists designed for observational studies ★★★★ = Excellent, rigorously addressed, clearly defined, ★★★ = Good, minor limitations identified, ★★ = Moderate, noticeable limitations in methodology, N/A = Criterion not applicable based on study type

Study	Criteria Clearly Defined	Subjects/Settings Described	Valid Exposure Measurement	Objective Criteria for Condition	Confounding Factors Identified	Strategies for Confounders	Valid Outcome Measurement	Appropriate Statistical Analysis	Overall Appraisal
Randive et al. [12]	★★★★	★★★★	★★★	★★★	★★★★	★★★★	★★★★	★★★★	Include (high quality)
Randive et al. [13]	★★★★	★★★★	★★★★	★★★★	★★★★	★★★★	★★★★	★★★★	Include (high quality)
Chaturvedi et al. [16]	★★★★	★★★★	★★★★	★★★★	★★★	★★★	★★★★	N/A (Qualitative)	Include (moderate quality)
Mishra et al. [24]	★★★★	★★★★	★★★★	★★★★	★★★★	★★★★	★★★★	★★★★	Include (high quality)
Chatterjee and Poddar [25]	★★★★	★★★★	★★★★	★★★★	★★★★	★★★★	★★★★	★★★★	Include (high quality)

**Table 6:** Critical appraisal of a study using the JBI Checklist for Systematic Reviews and Research Syntheses The Joanna Briggs Institute (JBI) tool was applied with modifications for the study by Nair and Panda [11] due to its distinct study design.

Study	Clearly Stated Review Question	Appropriate Inclusion Criteria	Appropriate Search Strategy	Adequate Sources and Resources	Appropriate Criteria for Appraisal	Independent Appraisal by Reviewers	Methods to Minimize Extraction Errors	Appropriate Methods to Combine Studies	Overall Appraisal
Nair and Panda [11]	★★★★	★★★	★★	★★★	★★★	★★	★★	★★	Include (moderate quality)

The results emphasize the program's achievements in boosting institutional deliveries and enhancing access to essential maternal and child health services, especially for marginalized groups. Nonetheless, there are considerable obstacles in ensuring fair access, addressing implementation shortcomings, reducing out-of-pocket expenses, and consistently impacting mortality rates.

The thematic analysis of the literature indicates that although the financial incentive strongly encourages institutional delivery, the program's success is shaped by a complex mix of factors, including the healthcare system's efficiency, socio-economic conditions, cultural practices, and the quality of care provided. Qualitative findings highlight the necessity of considering beneficiaries' lived experiences, who often encounter multiple barriers beyond financial limitations. The identified codes, such as 'financial accessibility', 'bureaucracy', 'hidden costs', and 'quality of care', offer a detailed understanding of the factors facilitating and hindering the program's success. Ensuring the availability of medications, round-the-clock access to skilled healthcare personnel, and avoiding unethical practices in service delivery will increase the willingness to utilize the facilities.

The variations in mortality outcomes observed across different studies indicate that merely increasing the number of institutional deliveries may not be enough to significantly lower maternal and neonatal death rates [34]. This underscores the urgent need for simultaneous enhancements in the quality of obstetric and neonatal care, which includes ensuring the presence of skilled birth attendants, the availability of essential medical supplies, and the effective management of complications.

When these findings are compared with the broader literature on CCTs in other low- and middle-income nations, similar trends of increased service use, especially for incentivized actions, are observed. Nonetheless, issues related to implementation, targeting, and sustaining impact are frequently noted.

To enhance the effectiveness of CCT programs, it is essential to make context-specific adjustments and engage in ongoing monitoring and evaluation [35]. Evidence suggests that financial incentives can improve both the availability and quality of maternal health services, while also addressing systemic obstacles that impede women's access to care and providers' capacity to offer crucial, life-saving maternal healthcare [36].

Implications for research and practice, and future scope of work

Future research must continue to adopt rigorous designs, such as quasi-experimental and longitudinal approaches, to robustly assess the causal impact of the JSSK’s CCT component on maternal and child health outcomes. A key priority should be evaluating the sustainability of behavioral shifts, especially institutional deliveries, after financial incentives are withdrawn. In-depth qualitative inquiries are equally essential to illuminate the lived experiences of beneficiaries, particularly in navigating access barriers and health system responsiveness.

An expanded analytical lens is necessary moving forward, one that considers the interactive influence of parallel and subsequent maternal-child health schemes. For example, the Kasturba Poshan Sahay Yojana (KPSY) [37] in Gujarat provides nutritional support to undernourished pregnant women and lactating mothers, and may indirectly enhance the uptake of institutional deliveries by improving antenatal health status and awareness of entitlements. Similarly, the Pradhan Mantri Matru Vandana Yojana (PMMVY) [38] offers maternity benefits for the first live birth, supporting rest and nutrition through CCTs. Such incentives, when coupled with those under JSSK, may amplify the effects on institutional delivery rates and postpartum recovery, complicating attribution but enriching the broader maternal health ecosystem.

More recently, the Namo Shree Yojana [39], launched in Gujarat, extends cash incentives for hospital deliveries among women with incomes under the BPL and from tribal communities, overlapping substantially with JSSK’s target groups. These schemes, while aligned in intent, introduce policy layering that warrants evaluation. Researchers should assess synergistic, additive, or potentially redundant effects of co-existing interventions, especially in high-focus states with multiple conditional benefit programs.

Moreover, implementation dynamics may be altered when schemes are introduced concurrently. For instance, transport support, free treatment, and diagnostics under JSSK may interact with KPSY’s nutrition supplementation and PMMVY’s wage compensation, leading to complementary benefits or administrative complexity. Future research must therefore account for this evolving policy landscape when evaluating maternal and neonatal outcomes such as mortality, morbidity, and quality of care received.

From a practice standpoint, program managers must prioritize inter-scheme convergence and streamlined beneficiary identification to reduce fragmentation and duplication. Strengthening frontline worker capacity (e.g., ASHAs, ANMs) to guide women through the multi-scheme environment is crucial for enhancing uptake and impact. Concurrently, technology-driven integration (e.g., a unified digital registry) can minimize leakages and facilitate real-time monitoring across schemes.

Lastly, cost-effectiveness evaluations comparing individual versus integrated program approaches will be vital to inform resource allocation. As maternal health policy continues to evolve with innovations like the Namo Shree Yojana, a comprehensive, systems-level evaluation framework will be essential to guide future program design and ensure equitable, high-quality maternal and child health services across India.

Recommendations

Based on the key findings of this narrative review, particularly those drawn from the SWOC analysis of the CCT component under JSY/JSSK, several recommendations are proposed to address implementation challenges, strengthen health system responsiveness, and support evidence-informed policy development. First, CCT incentives should be expanded to cover the full continuum of maternal care, including antenatal, intrapartum, and postnatal services, rather than focusing solely on institutional deliveries. Accountability mechanisms at the state and district levels must be reinforced to ensure timely disbursement of benefits, minimize leakages, and reduce out-of-pocket expenditures. Equitable outreach can be improved by refining beneficiary targeting and employing geospatial monitoring tools to better serve underserved populations. Additionally, the quality of care in public health facilities needs to be enhanced through continuous skill-building for healthcare providers, promoting respectful maternity care, and ensuring adequate infrastructural support.

It is also important to promote better integration of JSY/JSSK with other complementary schemes such as PMMVY, Kasturba Poshan Sahay Yojana, and Namo Shree Yojana, to achieve continuity of care and more efficient use of resources. Creating unified beneficiary databases and integrated digital platforms could further streamline access to multiple schemes and reduce administrative inefficiencies.

## Conclusions

The CCT component under India’s JSSK has played a pivotal role in increasing institutional deliveries and reducing access barriers, particularly among marginalized populations. However, findings from this narrative review highlight persistent challenges in implementation fidelity, equitable access, quality of care, and long-term outcome improvements. Notably, the effectiveness of the CCT scheme is influenced not only by its own design but also by concurrent schemes such as PMMVY, Kasturba Poshan Sahay Yojana, and Namo Shree Yojana, underscoring the need for convergence in maternal health policy. Enhancing program integration, addressing quality gaps, and incorporating a more holistic continuum-of-care approach are essential to achieve sustained reductions in maternal and neonatal morbidity and mortality. Future efforts must prioritize equity, accountability, and system-level improvements to realize the full potential of CCTs within India’s maternal and child health landscape.

## Appendices

Appendix 1

The search strategy is detailed in Table 7.

**Table 7:** Search strategy for different databases

Data base	Search strategy
PubMed	(((((Janani Shishu Suraksha Karyakram) OR (JSSK)) OR (Janani Suraksha Yojana)) OR (JSY) AND (2000:2025[pdat])) AND ((((((conditional cash transfer) OR (conditional cash transfers)) OR (CCT)) OR (incentive)) OR (financial benefit)) OR (benefit) AND (2000:2025[pdat]))) AND (India AND (2000:2025[pdat])) (("Janani"[All Fields] AND "Shishu"[All Fields] AND "Suraksha"[All Fields] AND "Karyakram"[All Fields]) OR "JSSK"[All Fields] OR ("Janani"[All Fields] AND "Suraksha"[All Fields] AND ("yojana"[Journal] OR "yojana"[All Fields])) OR "JSY"[All Fields]) AND 2000/01/01:2025/12/31[Date - Publication] AND (((("conditional"[All Fields] OR "conditionals"[All Fields]) AND "cash"[All Fields] AND ("transfer"[All Fields] OR "transferability"[All Fields] OR "transferable"[All Fields] OR "transfered"[All Fields] OR "transfering"[All Fields] OR "transferred"[All Fields] OR "transferring"[All Fields] OR "transfers"[All Fields])) OR (("conditional"[All Fields] OR "conditionals"[All Fields]) AND "cash"[All Fields] AND ("transfer"[All Fields] OR "transferability"[All Fields] OR "transferable"[All Fields] OR "transfered"[All Fields] OR "transfering"[All Fields] OR "transferred"[All Fields] OR "transferring"[All Fields] OR "transfers"[All Fields])) OR "CCT"[All Fields] OR ("incent"[All Fields] OR "incenting"[All Fields] OR "motivation"[MeSH Terms] OR "motivation"[All Fields] OR "incentive"[All Fields] OR "incentives"[All Fields]) OR (("economics"[MeSH Terms] OR "economics"[All Fields] OR "financial"[All Fields] OR "financially"[All Fields] OR "financials"[All Fields] OR "financier"[All Fields] OR "financiers"[All Fields]) AND ("benefit"[All Fields] OR "benefited"[All Fields] OR "benefiting"[All Fields] OR "benefits"[All Fields] OR "benefitted"[All Fields] OR "benefitting"[All Fields])) OR ("benefit"[All Fields] OR "benefited"[All Fields] OR "benefiting"[All Fields] OR "benefits"[All Fields] OR "benefitted"[All Fields] OR "benefitting"[All Fields])) AND 2000/01/01:2025/12/31[Date - Publication]) AND (("india"[MeSH Terms] OR "india"[All Fields] OR "india s"[All Fields] OR "indias"[All Fields]) AND 2000/01/01:2025/12/31[Date - Publication]) Translations Yojana: "Yojana"[Journal:__jid9878855] OR "yojana"[All Fields] conditional: "conditional"[All Fields] OR "conditional's"[All Fields] OR "conditionals"[All Fields] transfer: "transfer"[All Fields] OR "transferability"[All Fields] OR "transferable"[All Fields] OR "transfered"[All Fields] OR "transfering"[All Fields] OR "transferred"[All Fields] OR "transferring"[All Fields] OR "transfers"[All Fields] conditional: "conditional"[All Fields] OR "conditional's"[All Fields] OR "conditionals"[All Fields] transfers: "transfer"[All Fields] OR "transferability"[All Fields] OR "transferable"[All Fields] OR "transfered"[All Fields] OR "transfering"[All Fields] OR "transferred"[All Fields] OR "transferring"[All Fields] OR "transfers"[All Fields] incentive: "incent"[All Fields] OR "incenting"[All Fields] OR "motivation"[MeSH Terms] OR "motivation"[All Fields] OR "incentive"[All Fields] OR "incentives"[All Fields] financial: "economics"[MeSH Terms] OR "economics"[All Fields] OR "financial"[All Fields] OR "financially"[All Fields] OR "financials"[All Fields] OR "financier"[All Fields] OR "financiers"[All Fields] benefit: "benefit"[All Fields] OR "benefited"[All Fields] OR "benefiting"[All Fields] OR "benefits"[All Fields] OR "benefitted"[All Fields] OR "benefitting"[All Fields] benefit: "benefit"[All Fields] OR "benefited"[All Fields] OR "benefiting"[All Fields] OR "benefits"[All Fields] OR "benefitted"[All Fields] OR "benefitting"[All Fields] India: "india"[MeSH Terms] OR "india"[All Fields] OR "india's"[All Fields] OR "indias"[All Fields]
Embase	('janani shishu suraksha karyakram'/exp OR 'jssk'/exp OR 'janani suraksha yojana'/exp OR 'jsy'/exp) AND ('conditional cash transfer'/exp OR 'cash transfer'/exp) AND 'india'/exp AND (/py)
Google Scholar	"Janani Shishu Suraksha Karyakram" OR "JSSK" OR "Janani Suraksha Yojana" OR "JSY" "conditional cash transfers" OR "cash transfers" India (2009 OR 2010 OR 2011 OR 2012 OR 2013 OR 2014 OR 2015 OR 2016 OR 2017 OR 2018 OR 2019 OR 2020 OR 2021 OR 2022 OR 2023 OR 2024) (Note: Google Scholar's advanced search was used with these terms and the date range filter.)
